# A Case Report of Crohn’s Disease with Atypical Manifestations

**DOI:** 10.31661/gmj.v13i.3540

**Published:** 2024-10-15

**Authors:** Maedeh Esmailzadeh, Masoumeh Safaee

**Affiliations:** ^1^ Amin Hospital, Isfahan University of Medical Sciences, Isfahan, Iran; ^2^ Department of Surgery, School of Medicine, Isfahan University of Medical Sciences, Isfahan, Iran

**Keywords:** Crohn Disease, Abdominal Pain, Abdominal Mass, Postoperative, Surgery

## Abstract

Background: Crohn’s disease, a chronic inflammatory bowel disease, is characterized by inflammation of the gastrointestinal tract. While it typically manifests with abdominal pain, diarrhea, and weight loss, atypical presentations may arise, presenting diagnostic challenges. This case report aims to highlight the diagnostic challenges posed by atypical manifestations of Crohn’s disease. Case Presentation: A 31-year-old male sought medical attention in the Emergency Department due to drug-resistant abdominal pain and a distended abdomen. Following a thorough examination, including colonoscopy and surgical consultation, malignancy was suspected, prompting total colectomy and ileum-to-rectum anastomosis. Two large necrotic masses with purulent secretions were discovered in the cecum and sigmoid during the surgical procedure. Subsequent pathology results confirmed the diagnosis of Crohn’s disease, with no evidence of inflammation in other organs. The patient experienced a smooth recovery without surgical complications and was subsequently referred to a gastroenterology specialist for further management. Conclusion: This case emphasizes the importance of a multidisciplinary approach, including surgical and gastroenterology expertise, in the management and effective treatment of such cases in Crohn’s disease.

## Introduction

Crohn's disease (CD), one of the predominant inflammatory bowel diseases (IBD), exhibits a higher prevalence in developed nations. The incidence of CD is reported to be 0.1-0.6 cases per 100,000 patients annually, with an equal distribution among males and females [1][2]. Typically manifesting in early adulthood, CD presents with common symptoms such as abdominal pain, diarrhea, hematochezia, and weight loss [3]. Proposed etiology for the disease encompass diverse factors, including environmental influences, autoimmunity, genetic predisposition, and alterations in the gut microbiome. CD frequently exacerbates, leading to various complications, such as fistulas, abscesses, obstruction, and internal bleeding [4]. This case report aims to highlight the diagnostic challenges posed by atypical manifestations of Crohn's disease.

## Case Introduction

**Figure-1:**
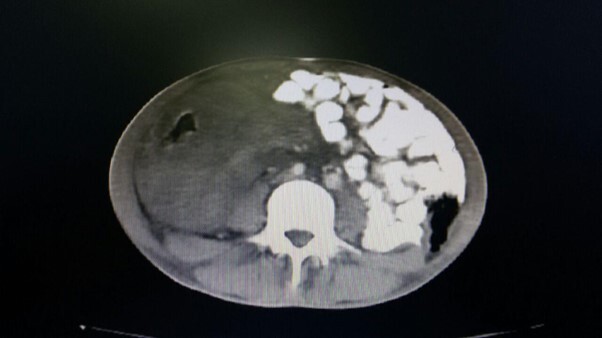
Figure 1. A CT scan of the patient before surgery indicated the size of the mass and the extent of abdominal involvement.

**Figure-2:**
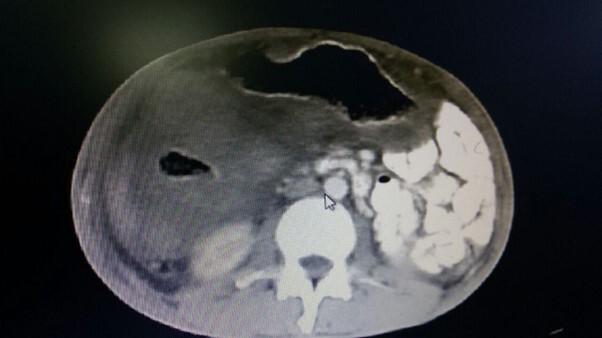
Figure 2. A CT scan of the patient before surgery indicated the size of the mass and the extent of abdominal involvement.

**Figure-3:**
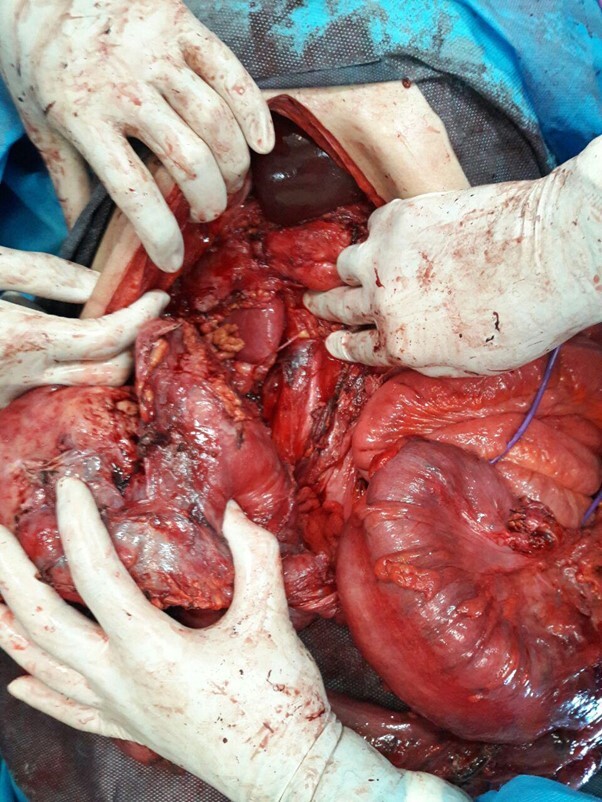
Figure 3. Photos during surgery. One mass measuring 220 x 180 mm, exhibiting severe adhesion to the liver, gallbladder, duodenum, and right kidney.

**Figure-4:**
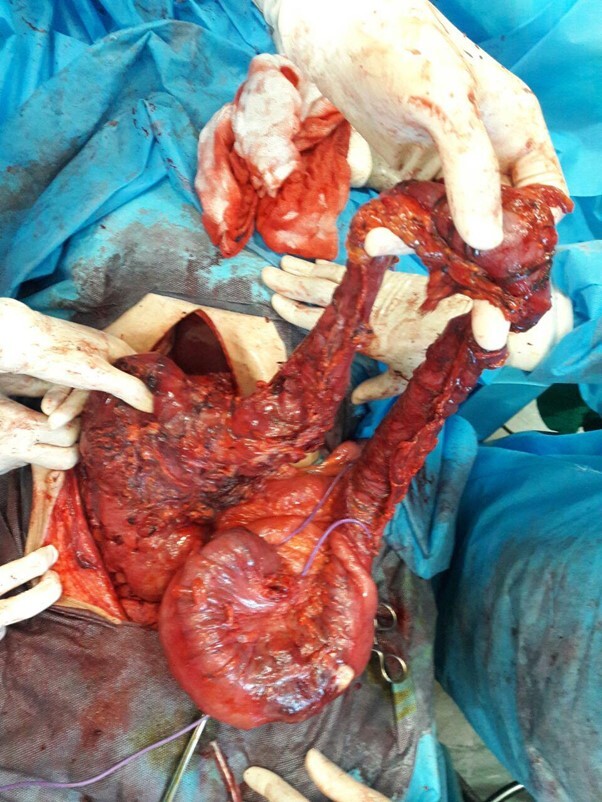
Figure 4. A photo taken during surgery (Another mass, measuring 150 x 100 mm, was located in the recto sigmoid region)

A 31-year-old male, an injection addict, presented with a gradual onset of abdominal pain, primarily localized in the right upper quadrant (RUQ) and right lower quadrant (RLQ), persisting for 15 days. Previous outpatient treatments had been administered for similar symptoms. This time, the patient experienced additional symptoms, including fever (39 degrees Celsius), chills, severe weakness, and lethargy. Initial admission to the infectious disease department was based on the manifestation of fever. Despite antibiotic treatment and ultrasound evidence, the patient showed no improvement, leading to a referral for a CT scan and subsequent evaluation by the surgery service.

In PMH, the patient has no previous history of any illness except drug addiction and then injecting them. No family history of Crohn's disease, IBD, or other digestive disorders. There is no family history of autoimmune disease or cancer. He has no history of previous surgery

Noteworthy is the absence of changes in bowel habits, such as bloody diarrhea or constipation. The patient reported a weight loss exceeding 10 kg over the last 3 months, accompanied by anorexia, but without vomiting. Physical examination revealed a thin and pale appearance, along with significant edema (+4) in the lower limbs and (+3) in the upper limbs. Palpation revealed a substantial mass and diffuse abdominal distention in the RUQ and RLQ. Other clinical examinations yielded normal results.

The conducted tests are outlined as follows:

WBC: 17300/mm3 NUT: 70.4% Hb: 7.4g/dl Plat: 1174000mm3 MCV: 79fl MCH: 24pg MCHC:30

. RDW: 3 g/dl/ ESR: 108mm /CRP: 68 (0-6) mg/d/ Alb: 2.1g/dl HCV Ab: Neg HIV Ab: Neg

HBs Ag: Neg/Na:136 mEq/ K: 4.2 mEq/ BUN: 14 mg/d Cr: 0.6 mg/d PTT: 28 Seco PT: 14.3

INR: 1.24 TIBC: 624 mcg/dl Amy: 25 U/I (NL) Lipase: 12 U/I (Nl)

Thyroid tests: normal. Liver tests: normal. (Except for ALK:921),

Blood culture on two occasions yielded negative results. A complete urine test exhibited normal findings, and serum iron and ferritin levels were within the normal range. Stool test for occult blood (OB) returned negative, and serum protein electrophoresis showed normal results. IgA antibody testing was negative, while tumor markers, specifically carcinoembryonic antigen (CEA), were elevated at 10 (considering the patient's smoking history).

Abdominal and pelvic ultrasound revealed slight free fluid in the anterior subhepatic space and mild free fluid in the pelvic region. A heterogeneous hypoechoic area with vascular flow, measuring 220 x 180 mm, was observed from the right subchondral area to the side.

In the CT scan, mild left-sided pleural effusion was noted, but increased thickness of the abdominal wall and irregularities in the wall of the small intestine, particularly in the terminal ileum, were evident. An enhancing soft tissue mass was observed on the side (Figure-1). The rightward displacement of the small intestine to the left side of the abdominal cavity was reported, along with free fluid in the abdomen and pelvis. (Figure-2).

Spleen heterogeneity raised suspicions, suggesting potential differential diagnoses of Gastrointestinal Stromal Tumors (GIST) and Carcinoid tumors. A hematology consultation was sought due to severe thrombocytosis, and a peripheral blood smear was performed, yielding normal results.

During colonoscopy, the ascending, transverse, and descending colons were reported as normal, with erythema and edema observed in the rectosigmoid area. Biopsies were taken, revealing no significant changes, but immunohistochemistry staining and T-Cell rearrangement ruled out lymphoma. The patient, deemed a candidate for laparotomy surgery, had undergone preoperative evaluation by a Urologist, revealing a mass in the ureter and right kidney.

Post-laparotomy, two masses were identified, one measuring 220 x 180 mm, exhibiting severe adhesion to the liver, gallbladder, duodenum, and right kidney. (Figure-3). Another mass, measuring 150 x 100 mm, was located in the rectosigmoid region (Figure-4).

The patient underwent total colectomy and ileum-to-rectum anastomosis utilizing circular stapler number 29. The pathology report from the colectomy revealed a mucous membrane exhibiting a cobblestone appearance with numerous longitudinal wound areas. Microscopic examination indicated infiltration of neutrophil-type inflammatory cells, a substantial presence of eosinophils, and a number of lymphoplasmacytic cells. No malignancy was detected, and these findings were more indicative of Crohn's disease (CD). The patient was subsequently referred to a gastroenterology specialist for further treatment.

As of now, approximately 6 months post-initiation of treatment, the patient's general condition is reported to be satisfactory. Anemia has been corrected, and there has been notable improvement in the patient's weight, anorexia, and abdominal pain.

This study was conducted in accordance with the fundamental principles of the Declaration of Helsinki. This study protocol was approved by Research Committee of Isfahan University of Medical Sciences. (Code number IR.ARI.MUI.REC.1402.157)

## Discussion

In this case report, a 31-year-old man, an injection drug addict, who presented with gradual onset of abdominal pain and continued for 15 days, was presented. Crohn's disease (CD) represents one of the two primary categories of intestinal inflammatory disorders [4]. While inflammatory bowel diseases, particularly CD, commonly manifest with symptoms like fever, abdominal pain, anemia, and digestive complications, the occurrence of the disease presenting initially with a substantial mass is a rare phenomenon, with limited reported cases [5]. Patients with inflammatory bowel disease (IBD) are at increased risk of developing gastrointestinal tumors, with adenocarcinoma being the most common and neuroendocrine tumor (NET) being the rarest [6]. In the majority of documented cases, the presence of a mass has been associated with malignancy [5]. Granulomatous masses occur in a number (but not all) of Crohn's patients due to the accumulation of inflammatory cells [7]. However, in the specific case under consideration, the identified mass exhibited evidence of granulomatous and purulent fistula, diverging from the usual malignant associations reported in existing literature. Inflammatory stasis cells are regarded as a plausible minimal causative factor for mass formation in CD [5]. Although most patients do not present with large masses during initial disease onset, this patient exhibited a notably large mass due to necrosis, contributing to the development of anemia [8]. According to the article by Guevara-Morales GR et al., in cases where intestinal resection is indicated due to intestinal stricture, side-to-side stapled anastomosis is recommended to reduce the risk of disease recurrence and stricture. In our recent patient, the ileum-to-rectum anastomosis was performed side-to-side with a ring stapler [9].

The unique presentation of a non-malignant mass in this case underscores the diverse and complex spectrum of manifestations within CD, emphasizing the importance of considering atypical presentations for accurate diagnosis and treatment planning.

## Conclusion

This case underscores the critical importance of recognizing the atypical presentation of Crohn's disease, which may closely resemble other medical conditions, thus posing diagnostic challenges. A quick and accurate diagnosis plays a fundamental role in timely and appropriate treatment initiation and prevents possible complications associated with delayed management of Crohn's disease. In this case report, the final pathology was Crohn's disease, despite the presence of symptoms of abdominal obstruction in an injection drug patient with a large abdominal mass. This suggests that clinicians considering Crohn's disease should maintain a high level of suspicion, even in patients with unusual symptoms without classic gastrointestinal manifestations.

## Acknowledgment

Not applicable.

## Conflict of interest

The authors declare that they have no competing interests.
